# Stochastic simulation to optimize rice breeding at IRRI

**DOI:** 10.3389/fpls.2024.1488814

**Published:** 2024-11-01

**Authors:** Fallou Seck, Parthiban Thathapalli Prakash, Giovanny Covarrubias-Pazaran, Tala Gueye, Ibrahima Diédhiou, Sankalp Bhosale, Suresh Kadaru, Jérôme Bartholomé

**Affiliations:** ^1^ Rice Breeding Platform, International Rice Research Institute, Metro Manila, Philippines; ^2^ Department of Crop Science, National Agricultural Institute (ENSA), University Iba Der Thiam of Thiès, Thiès, Senegal; ^3^ CIRAD, UMR AGAP, Cali, Colombia; ^4^ AGAP, Univ Montpellier, CIRAD, INRA, Montpellier SupAgro, Montpellier, France; ^5^ Crops for Nutrition and Health, International Center for Tropical Agriculture (CIAT), Cali, Colombia

**Keywords:** rice, genetic gain, stochastic simulations, genomic selection, breeding strategy

## Abstract

**Introduction:**

Genetic improvement in rice increased yield potential and improved varieties for farmers over the last decades. However, the demand for rice is growing while its cultivation faces challenges posed by climate change. To address these challenges, rice breeding programs need to adopt efficient breeding strategies to provide a steady increase in the rate of genetic gain for major traits. The International Rice Research Institute (IRRI) breeding program has evolved over time to implement faster and more efficient breeding techniques such as rapid generation advance (RGA) and genomic selection (GS). Simulation experiments support data-driven optimization of the breeding program toward the desired rate of genetic gain for key traits.

**Methods:**

This study used stochastic simulations to compare breeding schemes with different cycle times. The objective was to assess the impact of different genomic selection strategies on medium- and long-term genetic gain. Four genomic selection schemes were simulated, representing the past approaches (5 years recycling), current schemes (3 years recycling), and two options for the future schemes (both with 2 years recycling).

**Results:**

The 2-Year within-cohort prediction scheme showed a significant increase in genetic gain in the medium-term horizon. Specifically, it resulted in a 22%, 24%, and 27% increase over the current scheme in the zero, intermediate, and high genotype-by-environment interaction (GEI) contexts, respectively. On the other hand, the 2-Year scheme based on between-cohort prediction was more efficient in the long term, but only in the absence of GEI. Consistent with our expectations, the shortest breeding schemes showed an increase in genetic gain and faster depletion of genetic variance compared to the current scheme.

**Discussion:**

These results suggest that higher rates of genetic gain are achievable in the breeding program by further reducing the cycle time and adjusting the target population of environments. However, more attention is needed regarding the crossing strategy to use genetic variance optimally.

## 1 Introduction

Rice (*Oryza sativa* L.) provides sustenance for more than half of humanity. Low- and middle-income countries across the globe depend on rice as a primary dietary and nutrition source. By developing more productive and adapted varieties, genetic improvement has played a critical role in achieving higher rice production levels in smallholder fields (Prasad et al., 2017; Siddiq and Vemireddy, 2021). However, rice cultivation is facing important challenges as the population continues to grow in countries where rice consumption is high (Godfray et al., 2010; Ray et al., 2013; Tilman et al., 2011). Climate change makes growing conditions more difficult in these regions. Maximizing yield potential and resource use efficiency is crucial to overcoming these challenges (Siddiq and Vemireddy, 2021). Despite the important efforts of breeding programs, experts consider the levels of genetic gain achieved until now as low, failing to meet growing demand (Ray et al., 2013; Seck et al., 2023). As the breeding targets are becoming more advanced and complex, rice breeding programs need to develop high-yielding and adapted varieties that are more efficient, providing a steady increase in the rate of genetic gain in grain yield and other economically important traits (Cobb et al., 2019; Xu et al., 2021). Therefore, the optimization of breeding strategies is essential for breeders to increase the rate of genetic gain (Cobb et al., 2019; Rutkoski, 2019; Seck et al., 2023). This optimization dynamic potentially implies a wide range of scenarios that would be unrealistic to explore with field trials. Empirical testing of hypotheses is time- and resource-consuming. Simulation appears to be an interesting and reasonable alternative to test a wide range of hypotheses rapidly and at a low cost.

Owing to the development of high-performance tools for simulating breeding programs, breeders are increasingly using stochastic simulations to evaluate complex breeding strategies (Bančič et al., 2023; Gaynor et al., 2021; Li et al., 2012; Liu et al., 2019; Pook et al., 2020; Sun et al., 2011). In most cases, prospective studies are conducted to i) evaluate the performance of breeding schemes over the medium-to-long term, ii) compare several schemes, and iii) identify and guide the choice of the most effective breeding strategy. Simulations can help breeders guide their decisions. They can use simulations to choose and define the optimal number of crosses and progeny size (Covarrubias-Pazaran et al., 2022), the best genomic selection model, the extent of the target population of environments (Bančič et al., 2024), and a selection index, among other uses. Gaynor et al. (2017) demonstrated the effectiveness of using genomic selection (GS) in both single and two-part breeding strategies for inbred lines. Their study highlighted the advantages of implementing genomic selection, particularly in the early stages of the breeding process. Stochastic simulation was also used to evaluate the effectiveness of a two-part breeding program in clonal breeding. Parent selection based on genomic predicted cross-performance worked better than selection based on genomic estimated breeding values (GEBVs) (Werner et al., 2023). Cassava breeding simulations were used to evaluate the optimal number of parents along with the optimal number of crosses over two time horizons of 20 and 60 years (Covarrubias-Pazaran et al., 2022). Another approach — genetic complementation between heterotic genetic pools in a reciprocal recurrent selection context — also demonstrated the advantage of stochastic simulation (Covarrubias-Pazaran et al., 2023).

With its long history of innovation and important contribution to modern rice breeding, the International Rice Research Institute (IRRI) breeding program offers an interesting example of how breeding schemes can be optimized by integrating new knowledge and modern tools. IRRI’s breeding program began in 1960 with the main objective of addressing the food crisis in Asia by developing high-yielding, fertilizer-responsive, and lodging-resistant rice varieties (Peng and Khushg, 2003). Short-statured plants were ideal for combating the lodging problem. Breeders at IRRI created IR8, the first high-yielding, semi-dwarf variety. IR8 was developed using the pedigree method and visual selection, the most common approaches used at that time. With the evolution of demands and constraints related to rice production, breeding targets became more complex, focusing on higher yield potential, grain quality, disease and insect resistance, and other traits of economic importance (Ali et al., 2021; Prasad et al., 2017; Siddiq and Vemireddy, 2021). This new context led to the integration of more information via multi-environment testing and more tools, such as breeding informatics or high-throughput genotyping (Cobb et al., 2019, 2018; Xu et al., 2017). However, for several decades, the main approach to developing inbred varieties remained the pedigree method, even if it implied a long breeding cycle (around 8 - 10 years). To overcome this limitation, IRRI’s breeding program has recently initiated an optimization process in its strategy to develop modern rice varieties. The approach incorporates faster breeding techniques to shorten the breeding cycle time and, therefore, increase the rate of genetic gains. Initially, the new breeding strategy was focused on the rapid fixation of segregating material through single seed descent using rapid generation advance (RGA) techniques to reduce costs and time to fixation. RGA was designed to take place in the greenhouse on a seedling plate, under artificial short days, at high temperatures, allowing up to four generations a year (Beredo et al., 2016; Collard et al., 2019, 2017). RGA reduced the breeding cycle to about 6 or 7 years. Taking advantage of the rapid fixation of the lines and low-cost molecular markers, the program then implemented routine marker-assisted selection for major disease-resistance genes. The objective was to quickly increase the frequencies of critical alleles in the program (Cobb et al., 2018). The last main evolution in the breeding scheme was integrating genomic selection (GS) for population improvement, within a closed system and based on elite-by-elite parental crosses (Juma et al., 2021; Khanna et al., 2022). Using genomic information in multi-environment evaluation for within-cohort prediction helped to increase the selection intensity and accuracy on traits like grain yield. In this context, a “cohort” refers to a group of lines treated as a unit and generated from a set of crosses in each breeding cycle (parallel breeding cycles). The integration of GS enabled the program to decrease the cycle time to 5 years (Bartholomé et al., 2022).

In the past, breeding programs at IRRI evaluated early-stage breeding lines mainly at one location. The best-performing lines were then selected and distributed to national agricultural research partners for further evaluation in late-stage yield trials. However, this tactic had limitations. The approach reduced the likelihood of identifying suitable lines for national-level nominations at a particular target population of environments (TPE) because the early-stage evaluation does not happen in the TPE. Moreover, the small proportion of best-performing breeding lines produced by this strategy tends to be stable and broadly adapted. In recent years, IRRI has changed its evaluation strategy. Now, advanced yield trials are conducted in the regions by the partners. This approach, facilitated by the expansion of the breeding network, harnesses data from multiple locations within countries to inform breeding decisions. Utilizing such data is crucial for identifying superior genotypes within the TPE and for ensuring the development of stable, high-yielding cultivars for farmers across diverse environments (Bančič et al., 2024; Tolhurst et al., 2022). However, achieving comprehensive coverage of the TPEs poses challenges, particularly when resources limit the number of testing locations and years. In that case, the breeder needs to deal with the available environments and the underlying genotype-by-environment interactions (GEI). Identifying GEI, which reflects differential genotype responses across environments or shifts in performance ranks, requires integrated approaches to genotype evaluation across multiple environments (Malosetti et al., 2013; Zakir, 2018). For complex traits controlled by many genes, like grain yield, GEI is usually considered to represent an important proportion of the total phenotypic variance (Cooper et al., 1999; Cullis et al., 2000). GEI’s importance impacts the evaluation strategy as well as the prediction of the phenotypic performance in the case of a strategy based on GS. Recently, IRRI scientists have worked to better integrate the information of multi-environment trials (MET) and take into account GEI in the context of genomic predictions (Nguyen et al., 2023). Results have shown that the typical level of GEI relative to the genetic main effect variance (GEI:main) encountered for grain yield was of the order of 792:296.

In this context, sustainable yield improvement in rice depends on understanding the impacts of changes in the breeding strategies. Through simulation experiments, this study aims to evaluate cycle shortening and GS effects on medium- and long-term genetic gain and the efficacy of future strategies for the IRRI breeding program. The analysis uses different GS methods (within *vs*. between cohort prediction) to compare past, present and future strategies to achieve high rate of genetic gain. We seek to identify optimal strategies to maximize genetic gain and efficiently use the available genetic variability. The efficiency of the different strategies was compared under different levels of GEI, which reflect the mega-environments in which the breeding program operates with its partners. Such insights are crucial for guiding the future direction of rice breeding programs, particularly in the face of escalating challenges posed by climate change and increasing demand.

## 2 Materials and methods

### 2.1 Breeding schemes

**Figure 1:**
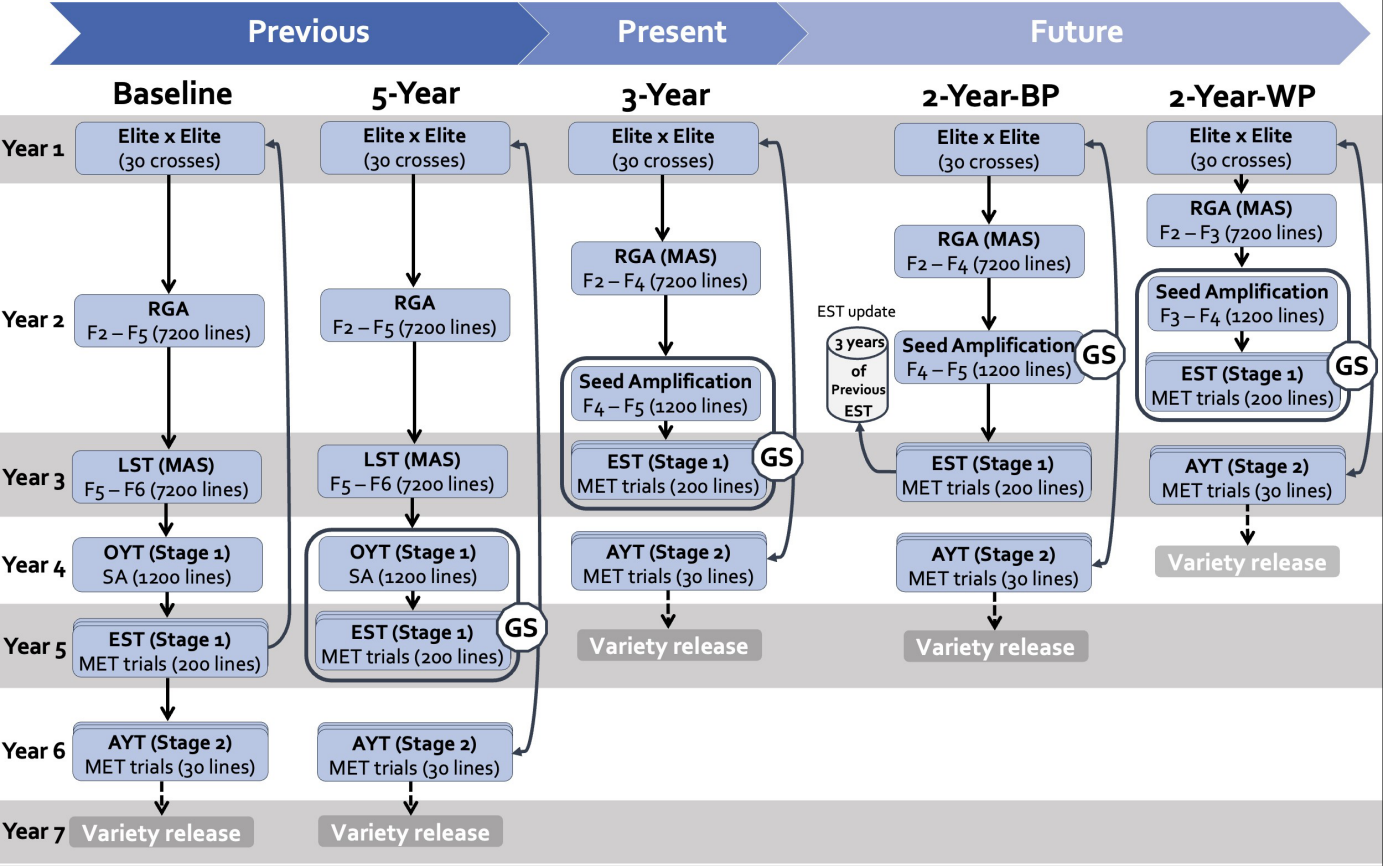
Graphical representation of the breeding schemes simulated in this study. In columns, the different schemes are presented as follows: Baseline (phenotypic selection); 5-Year parent recycling scheme; 3-Year parent recycling scheme, the 2-Year between-cohort prediction recycling scheme (2-Year-BP), and the 2-Year within-cohort prediction recycling scheme (2-Year-WP). RGA, Rapid Generation Advancement; LST, Line Stage Testing; OYT, Observational Yield Trial; SA, Seed Amplification; EST, Estimation Set) AYT, Advanced Yield Trial; GS, Genomic Selection; MET, Multi Environment Trial.

The IRRI breeding strategy for a transplanted medium-maturity rice breeding pipeline (former irrigated breeding program) has evolved from classical pedigree breeding with phenotypic selection to GEBV-based GS (**Figure 1**). Integrating GS, in conjunction with other tools, has helped reduce cycle time due to the higher accuracy of the merit surrogates, allowing earlier decision-making. In this study, we compared past, present, and potential future breeding schemes using simulation. We evaluated the impact of shortening the breeding cycle (earlier parental selection) on achieving high genetic gain and other breeding-program performance indicators. The past, present and future breeding strategies are described below. The breeding schemes are named according to the cycle length.

#### 2.1.1 Previous breeding scheme: 5-Year parent recycling scheme

The 5-Year parent recycling scheme is based on RGA and genomic selection. The strategy involves two stages of multi-location yield evaluation to select elite breeding lines at the F9 or F10 generation (**Figure 1**). The scheme is designed as a closed system. The recycling of the elite lines as parents for the subsequent cycle takes place in Year 5 (Stage 1 yield trials) based on genomic estimated breeding values (GEBVs). The main steps are described below.

**Crosses (Year 1):** At the beginning of each cycle, 40 unique elite lines are selected as parents. The crossing plan would be based on a half-diallel; however, given the large number of combinations, only 30 crosses are selected based on the coefficient of parentage between crossed lines, the average breeding values for grain yield, the appropriate maturity, and the frequency of major QTLs (biotic and abiotic resistance, grain quality).

**RGA (Year 2):** Breeders employ the single seed descent (SSD) method to establish F2 families for line testing and yield evaluation. This rapid fixation process, known as rapid generation advance (RGA), allows for the development of four generations in a single year. The process generates 240 F6 lines from each selected cross, resulting in 7,200 lines.

**Line testing and marker-assisted selection (Year 3):** This step controls the uniformity of the fixed lines (contamination or cross-pollination) and discards lines susceptible to diseases and stresses. The 7200 F6 lines from the RGA system are evaluated in head-rows (one line per row). During this step, called line stage testing (LST), marker-assisted selection (MAS) identifies materials with disease resistance (blast and bacterial blight), along with additional selections for other agronomic traits such as earliness, plant height, and grain quality. The selection is limited to within-family selection. Forty lines per family are advanced, reducing the number of lines to 1,200 for the first-stage yield trial.

**Stage 1 of yield evaluation (Year 4):** An initial yield trial of the 1,200 lines is carried out on large plots in a single season. The purpose of this trial is mainly seed amplification for METs in different target breeding zones. These 1200 lines are for observational yield trials (OYT). The 1,200 OYT lines are also genotyped using SNP markers of the 1k-RiCA (Arbelaez et al., 2019).

**Stage 1 training set (Year 5):** A within-cohort prediction is performed to select parents for subsequent cycles and advancement for Stage 2 yield trials. For this purpose, a representative subset of 200 genotypes (covering all crosses) is retrieved from the 1,200 lines. This training population, coded EST (estimation set), is shipped to regional partners and evaluated in METs in four locations in one season, with two replications. These data are used to train the GS model and predict the GEBVs of the 1,200 OYT lines. The GEBVs of Stage 1 are then used to select parents for the next cycle and line advancement in the Stage 2 yield trial.

**Stage 2 of yield evaluation (Year 6):** A set of 30 lines is selected as future products from OYT lines based on GEBVs. To acquire more precise genetic values, these selected lines are submitted to a second stage of yield trials in the different breeding zones. They are evaluated in four locations in one season, with two replicates in each field trial. This step, called the advanced yield trial (AYT), ends the breeding cycle and evaluates the performance of the final lines. The process requires about six years to develop high-performing lines, which are then submitted to multi-location trials in national trial systems for variety release in subsequent years.

#### 2.1.2 Current breeding scheme: 3-Year parent recycling scheme

The 3-Year recycling scheme is derived from the 5-Year scheme framework, with an early exit of the line fixation stage at the F4 generation (**Figure 1**). The 7,200 lines are reduced to 1,200 lines through the MAS in the second season of Year 2 and advanced to the F5 generation, followed by a seed amplification step for Stage 1 yield trials. In Year 3, a training set of 200 lines selected from the 1,200 lines based on markers is shipped to regions to be phenotyped in MET yield evaluations in four locations in each region. The second season of Year 3 is allotted to the within-cohort prediction of the breeding values of the 1,200 lines. The GEBVs are used to select lines that integrate the next cycle as parents and lines for advancement in the AYTs of Stage 2 in Year 4, which follows the same process as the 5-Year scheme. The LST and OYT stages are dropped, hence reducing the time from crossing to parent’s recycling by two years and the product development cycle to four years.

#### 2.1.3 Future schemes: 2-year parent recycling schemes

Alternative breeding schemes (**Figure 1**) were designed using the previous scheme’s template and focused mainly on shortening the breeding cycle length.

The 2-Year within-cohort prediction (2-Year-WP) is an upgrade of the previous 3-Year scheme by reducing the RGA exit at the F3 generation, followed by MAS to reduce the 7200 genotypes to 1,200 genotypes. The 1,200 F3 genotypes are advanced to F4 through the seed amplification stage during the first season of Year 2. Stage 1 of the METs is carried out during the second season of Year 2. The training set sample of Stage 1 followed the same method as in previous breeding schemes. The GEBVs of the 1,200 lines after Stage 1 were used both to select parents for the next cycle in Year 2 and line advancement in the Stage 2 yield trial in Year 3.

The 2-Year between-cohort prediction (2-Year-BP) is similar to the 3-Year scheme with a shift to between-cohort GEBVs prediction of untested genotypes (predicted population) based on previous data (training population). The between-cohort prediction enables the estimation of the GEBVs of the 1,200 lines in Year 2 rather than waiting for data from the training set in Year 3. To do so, an initial training population is built by gathering data from the Stage 1 MET yield trials of the last three training sets. The parents of the next breeding cycle and the line advancement to Stage 2 are selected based on the GEBVs in Year 2. In Year 3, a training set is selected from the 1,200 lines and evaluated on MET trials across the four locations in each breeding zone. The data from the current training set is included in the training set for the next cycle prediction and so on. Line advancement in Stage 2 for product development is carried out in Year 4. Indeed, the 2-Year-BP recycling scheme follows the same operations as the 3-Year cycle scheme, but the parental selection decision occurs in Year 2.

#### 2.1.4 Baseline: a reference based on phenotypic selection

We have included a baseline scheme in our study as a reference, corresponding to the 5-Year scheme without GS. In this approach, parent selection and advancement are based solely on phenotypic data. Parents for the next cycle are selected from the equivalent of Stage 1 to maintain the same recycling length. The baseline indicates the impact of genomic selection on genetic performance.

### 2.2 Simulation approach

The AlphaSimR program (CRAN - Package AlphaSimR) was used to perform the stochastic simulations of the rice genome structure (ancestral haplotypes), the genetic architecture of the trait of interest, and the breeding scheme (Gaynor et al., 2021). The comparison between schemes was based on 100 iterations for each scheme with the same initial burn-in scheme. Considering the relative complexity of some breeding operations and the structure of AlphaSimR, we adopted the following assumptions about the breeding process: (i) the 30 crosses from the half-diallel design are randomly selected; (ii) as a single trait is being simulated (grain yield), MAS step is simulated through phenotypic selection with low heritability; (iii) the training set is also randomly selected. All these actions are equally applied in all breeding scenarios.

#### 2.2.1 Base population establishment: burn-in

A burn-in phase begins with phenotypic selection to establish a common starting point for the evaluated breeding schemes. The burn-in scheme was similar to the baseline scheme with a reduced selection pressure and multi-cohort recycling. Eighty non-inbred founders are generated based on the parameters described in **Table 1**. To initiate the first cycle, 100 crosses are made from the 80 non-inbred individuals. The 12,000 lines are then advanced to the F6 generation. Two thousand five hundred lines are selected from the LST stage and advanced to the OYT stage. After phenotyping in OYT, 600 lines are selected and evaluated in Stage 1, from which 50 lines are selected and evaluated in the last Stage 2 trial. All the selections are based only on phenotypic values. The 80 parents for the next cycle are selected from the last two yield trial stages based on phenotypic performance. The burn-in was run over 40 years from the founders’ population. Then, each evaluated breeding scheme was simulated independently over 30 years from the same base population derived from the last burn-in cycle.

**Table 1:** Simulation features.

Phase		Parameters
Burn-in	Genome sequence	12 chromosome pairs 1.20 Morgans per chromosome 3 × 10^7^ base pairs per chromosome
	Founder genotypes	80 non-inbred founders Effective population size = 60 3000 QTLs (additive and GEI effects) Normally distributed QTL effects Genetic mean = 0 and Genetic variance = 1. GEI variance = c(0, 4, 8) H^2^_Plant = 0.001 and H^2^_Plot = 0.10
	Recent phenotypic breeding	40 years of conventional phenotypic breeding from the founder population
Evaluation	Breeding schemes	30 years of future breeding. Testing alternative breeding schemes according to the recycle time and integration of genomic selection

#### 2.2.2 Genomic structure and trait modeling

The genome of 12 chromosomes was simulated based on the information on the rice genome (Eckardt, 2000; Jackson, 2016). The founder haplotype sequences, including the 12 chromosomes, were simulated using the Markovian Coalescent Simulator (MaCS) algorithm implemented in AlphaSimR, assuming a diploid genomic structure, a chromosome length of 1.20 morgan, and 3x10^7^ base pair (bp), and an effective population size of 60, representing the effective size of the non-elite genetic materials (**Table 1**). The mutation rate is set to 2.5x10^-8^/bp.

A single polygenic trait representing grain yield was simulated for all breeding schemes. Only additive and genotype-by-environment interaction effects were modeled since dominance is less relevant for inbred lines. This genetic architecture was simulated by assuming 3,000 QTLs equally distributed across chromosomes. The genetic value of each individual is modeled by the following equation (Gaynor, 2023):


(1) $$GV=\;\mu +\sum_{1}^{nQTL}ax_{A}+w\;*\sum_{1}^{nQTL}ɡx_{A}$$

where GV is the genetic value of an individual; μ is an intercept, representing the mean genetic value parameter defined in the founder population and set to 0; the additive effect of the total QTL is a summation over all QTL for the product of the additive effect ( a ) and the scaled additive dosage vector ( $x_{A}$ ). The additive effect of each QTL value is sampled from a standard normal distribution and scaled to achieve the genetic variance $\sigma_{A}=1$ , defined in the founder population. The scaled additive genotype dosage scales the relative allele dosage {-1; 0; 1} to set the values for opposing homozygotes to -1 and 1, and the heterozygote values to 0. The genotype-by-environment interaction effect of each QTL represents a product of an environmental covariate effect ( w ) and a genotype-specific slope (genetic component). The environmental covariate, which represents the random environmental component of the GEI, was sampled each year from a normal distribution. The genotype-specific slope is modeled as a summation over all QTLs for the product of a GEI effect ( ɡ ) and the scaled additive dosage ( $x_{A}$ ). The GEI effects are sampled from a normal distribution with a mean of zero and a variance equal to the defined genotype-by-environment interaction variance.

The phenotypic value was modeled as the sum of the GV and an environmental deviation. Environmental error deviates are sampled from a normal distribution with a mean equal to 0 and a variance equal to the defined environmental error variance. Therefore, the precision of phenotyping relied mainly on environmental error variance. The genetic variance was set to 1, and two levels of environmental variance were set according to the stages of the field trials. The two levels of the environmental variance are defined as corresponding to (i) a row heritability of 0.001 on LST and seed amplification stages (ii) and a plot heritability of 0.10 on MET yield trials. The GEI variance was set to three levels to assess the effect of GEI interaction on breeding performance and to highlight the importance of the TPE definition. The approach defined GEI variances of 0, 4, and 8, corresponding to null, intermediate, and high genotype-by-environment interaction, respectively (**Supplementary Table S1**).

#### 2.2.3 The estimation of genomic breeding values

To mimic genotyping using the 1k RiCA platform, 1,200 SNP markers uniformly distributed across the 12 chromosomes were simulated. The information was used for applying genomic selection for parental selection and for line advancement in Stage 2. The prediction of the GEBVs for line advancement and parent selection at Stage 1 was performed by using the Ridge Regression Best Linear Unbiased Prediction (RR-BLUP) model for all breeding schemes according to the following model:


(2) y=1β+Zu+ϵ

where y is an (*n × 1*) vector of trait phenotypes; β is a vector fixed effect; 1 is a (*n x 1)* vector of 1; u is an (*m x 1*) vector of marker effects; Z is an (*n x m*) design matrix containing the genotypes of *n* lines for *m* biallelic SNP markers, coded as {-1,0,1}; ϵ is a vector of residuals. The u and ϵ vectors are assumed to be random.

The RR-BLUP model for genomic prediction is fitted using the *RRBLUP* function from the AlphaSimR package.

In the 2-Year-WP, 3-Year, and 5-Year recycling schemes, the GEBVs estimation of Stage 1 for line advancement and parent selection is performed based on data from the training population available in years 2, 3, and 5, respectively. In each cycle, a new training set of 200 lines was randomly selected, including all families. Unlike the 2 Year-BP scheme, the genomic prediction in the first cycle was based on an initial training population selected from the last three years of yield evaluations in the burn-in phase, consisting of a total of 600 lines. After the first cycle, a subset of 200 lines, randomly selected from the 1,200 lines and including all families, is evaluated on MET trials and added to the training population for prediction of the next cycle, and so on.

#### 2.2.4 Breeding schemes comparison

Realized genetic gain, prediction accuracy, genetic variance, and frequency of the favorable alleles made up the four performance indicators assessed in this analysis. The realized genetic gain (ΔG) was estimated as the regression coefficient of the mean genetic value of Stage 1 against the breeding years by fitting the following model:


(3) $$Y_{i}=\mu +\beta x_{i}+\delta_{i}$$

where $Y_{i}$ is the mean genetic value of year i ; μ is the intercept; β is the linear regression coefficient representing the rate of genetic gain per unit and year; $x_{i}$ represents the breeding year; and $\delta_{i}$ is the deviation from the linear model.

The average of the true genetic value for each replicate was centered on zero in Year 0 as the difference between the average of the true genetic value of the Stage 1 lines in each cycle and the average of the true genetic value in Year 0 corresponding to the base population. Two-time horizons were compared to assess genetic gain: the first 15 years (medium-term) and all 30 years (long-term).

The evolution of the additive genetic variation over the breeding cycle was also tracked at Stage 1, as well as the prediction accuracy and the frequency of the favorable allele in selected parents. The prediction accuracy was calculated as the correlation coefficient between the GEBVs and the true genetic values. For the baseline, since the selection is made only on phenotypic performance, the accuracy was estimated as the correlation with the true genetic values.

## 3 Results

### 3.1 Realized genetic gain among breeding schemes

**Figure 2:**
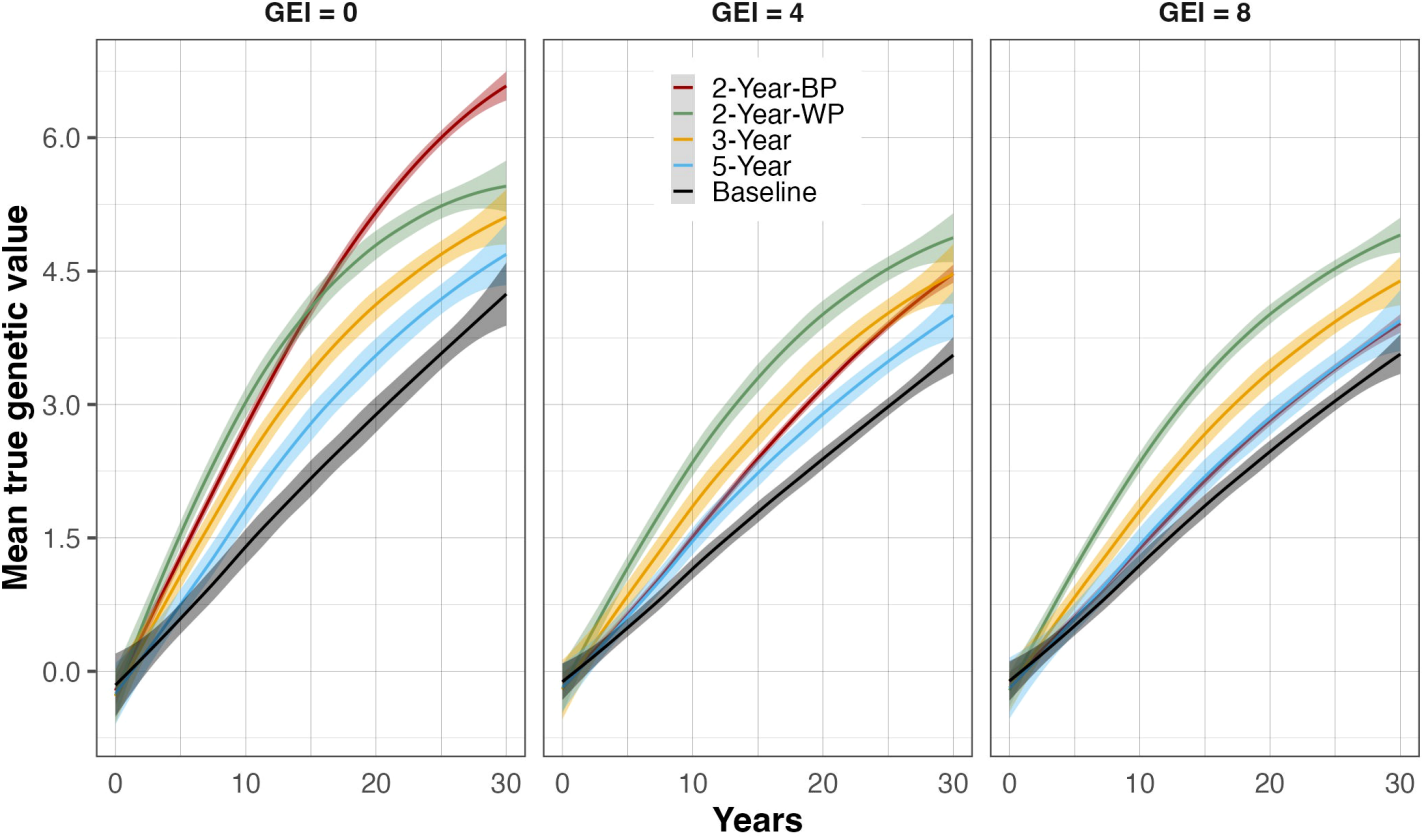
True genetic value trends at the parent-recycling stage over 30 breeding years for the four breeding schemes and the Baseline. Three levels of genotype-by-environment interaction (GEI) variance are represented: 0 (left panel), 4 (middle panel), and 8 (right panel) times greater than the main genetic variance. The breeding schemes are represented as colored lines: the 5-Year parent recycling scheme (5-Year), 3-Year parent recycling scheme (3-Year), the 2-Year between-cohort prediction recycling scheme (2-Year-BP), and the 2-Year within-cohort prediction recycling scheme (2-Year-WP). The solid lines represent the average value, and the shaded areas represent the associated standard error based on 100 replicates for each scenario.

The true genetic values of the breeding population showed a continuous increase over the 30 years of selection for all the breeding schemes, resulting in positive genetic gains (**Figure 2**; **Table 2**). The baseline strategy was the lowest-performing scheme, regardless of the GEI level and the breeding horizon, achieving an annual rate of genetic gain of 1.04% and 1.08% for intermediate (4) and high (8) GEI levels, respectively, and a maximum annual genetic gain of 1.29% when GEI was 0 (**Table 2**). Incorporating GS without changing the cycle length improved the realized genetic gain compared to the baseline. Indeed, the 5-Year scheme delivered a rate of genetic gain of 1.69%, 1.35%, and 1.33% for GEI levels 8, 4, and 0, respectively. The breeding strategies with shorter recycling time (two or three years) had the highest rates of genetic gain, ranging from 1.25 to 2.41% in the medium-term (15 years) and 1.08 to 1.83% in the long-term (30 years), depending on the level of GEI (**Table 2**). As expected, the rates of genetic gain decreased as the intensity of GEI increased (**Figure 2**; **Supplementary Figures S1**, **S2**), but the sensitivity of the breeding strategies relative to GEI levels varied.

**Table 2:** Genetic gain per year and rate of genetic gain per year for all breeding schemes.

Scheme	GEI	Medium-Term (15 years)		Long-Term (30 years)	
		Gain per year (± SE)	Rate per year (%)	Gain per year (± SE)	Rate per year (%)
2-Year-BP	0	0.289 ± 0.0054	2.36	0.233 ± 0.0044	1.83
2-Year-WP		0.295 ± 0.0053	2.41	0.186 ± 0.004	1.46
3-Year		0.241 ± 0.0038	1.97	0.18 ± 0.0032	1.41
5-Year		0.207 ± 0.003	1.69	0.169 ± 0.0026	1.32
Baseline		0.158 ± 0.0026	1.29	0.148 ± 0.0026	1.16
2-Year-BP	4	0.171 ± 0.0067	1.39	0.16 ± 0.0045	1.25
2-Year-WP		0.236 ± 0.0065	1.92	0.168 ± 0.0039	1.32
3-Year		0.191 ± 0.005	1.56	0.158 ± 0.003	1.24
5-Year		0.165 ± 0.0048	1.35	0.142 ± 0.0031	1.11
Baseline		0.128 ± 0.0034	1.04	0.124 ± 0.0024	0.97
2-Year-BP	8	0.153 ± 0.0074	1.25	0.138 ± 0.005	1.08
2-Year-WP		0.238 ± 0.0065	1.94	0.169 ± 0.0042	1.32
3-Year		0.187 ± 0.005	1.53	0.154 ± 0.0035	1.21
5-Year		0.163 ± 0.0049	1.33	0.14 ± 0.0033	1.10
Baseline		0.133 ± 0.0041	1.08	0.125 ± 0.0028	0.98

Three levels of genotype-by-environment interaction (GEI) variances are compared: 0, 4, and 8. The different breeding schemes are: 5-Year parent recycling scheme (5-Year), 3-Year parent recycling scheme (3-Year), 2-Year between-cohort prediction recycling scheme (2-Year-BP), and 2-Year within-cohort prediction recycling scheme (2-Year-WP).

When comparing the 2-Year-WP (within cohort) and 2-Year-BP (between cohorts), we observed that the latter was more sensitive to GEI, resulting in a strong reduction in genetic gain as the level of GEI increased. On the other hand, the ranking order of 2-Year-WP, 3-Year, and 5-Year schemes, as well as the baseline, remained constant irrespective of the level of GEI. When considering the medium-term, the 2-Year-WP was the best-performing breeding scheme at all GEI levels, followed by the 2-Year-BP, the 3-Year, and the 5-Year scheme. The 2-Year-WP was up to 28% and 46% better than the 3-Year and 5-Year schemes, respectively (**Table 3**). For the 2-Year-BP, the gain over the 3-Year and the 5-Year schemes was highly dependent on the level of GEI. At GEI = 0, the increase in genetic gain was 20% and 40% relative to the 3-Year and 5-Year schemes, respectively. However, this advantage dropped to -18% and -6% for the higher levels of GEI. When considering the long-term, the advantage of the 2-Year-WP was reduced. It ranged from 3% to 10% compared to the 3-Year scheme and from 10% to 21% compared to the 5-Year scheme (**Table 3**). Contrary to 2-Year-WP, the 2-Year-BP performed better in the long term with an increase of -10% to 29% and of -1% to 38% compared to the 3-Year and 5-Year schemes, respectively. Contrary to the 2-Year-WP, the advantage of the 2-Year-BP was higher when the level of GEI decreased.

**Table 3:** Genetic gains for 2-Year-BP and 2-Year-WP schemes relative to the 3-Year and 5-Year schemes, expressed as a percentage.

Scheme	GEI	Medium-Term (15 years)		Long-Term (30 years)	
		3-Year	5-Year	3-Year	5-Year
2-Year-BP	0	19.92	39.61	29.44	37.87
	4	-10.47	3.64	1.27	12.68
	8	-18.18	-6.13	-10.39	-1.43
2-Year-WP	0	22.41	42.51	3.33	10.06
	4	23.56	43.03	6.33	18.31
	8	27.27	46.01	9.74	20.71

The three levels of genotype-by-environment interaction (GEI) variance are presented.

### 3.2 Accuracy of genomic predictions

**Figure 3:**
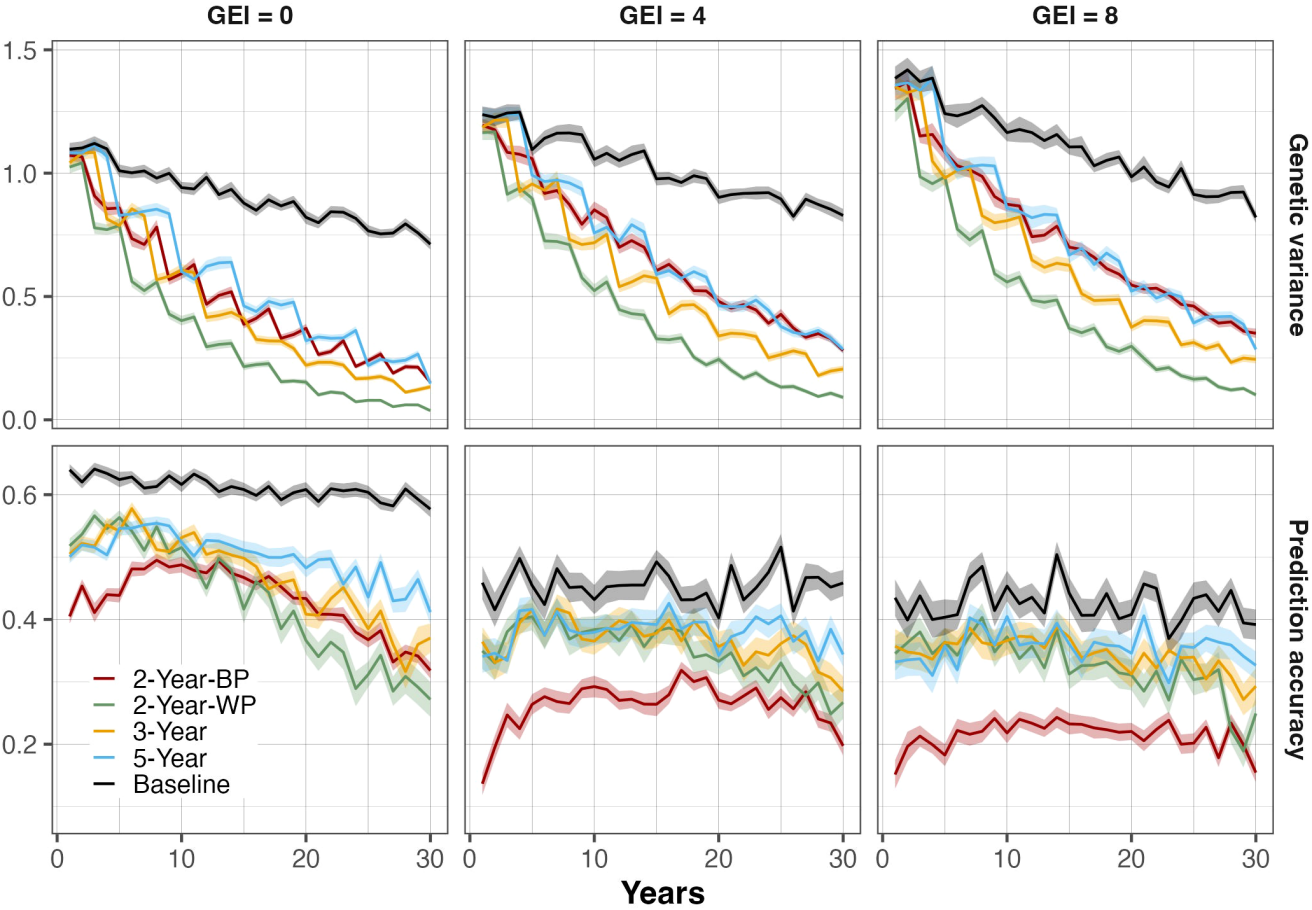
Evolution of genetic variance (top panels) and genomic prediction accuracy (bottom panels) at the parent-recycling stage over 30 breeding years for all breeding schemes. Three levels of genotype-by-environment interaction (GEI) variance are represented: 0 (left panel), 4 (middle panel), and 8 (right panel). The colored lines represent the different breeding schemes and the Baseline: the 5-Year parent recycling scheme (5-Year), 3-Year parent recycling scheme (3-Year), the 2-Year between-cohort prediction recycling scheme (2-Year-BP), and the 2-Year within-cohort prediction recycling scheme (2-Year-WP). The solid lines represent the average value, and the shaded areas represent the associated standard error based on 100 replicates for each scenario.

Considering all the scenarios, the accuracy of the genomic predictions ranged from 0.27 to 0.58 in the absence of GEI (**Figure 3**; **Supplementary Table S2**). As the level of GEI increased, the accuracy logically decreased and ranged from 0.14 to 0.43 for intermediate GEI and from 0.12 to 0.41 for high GEI. Accuracies tended to decrease over time, but the magnitude was related to the scheme and the GEI level. Indeed, the decline was more pronounced for the schemes with the shortest breeding cycle (2-Year-WP, and 2-Year-BP). The 2-Year-BP displayed the lowest prediction accuracy in all GEI scenarios. The 2-Year-BP accuracy showed a marked increase during the first couple of years before declining continuously. It reached a maximum accuracy of 0.49, 0.32, and 0.24 in absent-, intermediate-, and high-GEI, respectively. In the absence of GEI, the accuracy for the 2-Year-WP decreased from 0.52 to 0.27 in Year 30 (-48%), while the accuracy for 2-Year-BP decreased from 0.40 to 0.32 (-32%). In the presence of GEI, the reduction in accuracy was less important (**Figure 3**; **Supplementary Table S2**). The accuracy for 3-Year schemes was more stable, with a 20 to 26% reduction for the high level of GEI and in the absence of GEI, respectively. A similar trend was observed for the 5-Year scheme when GEI was absent, displaying 18% reduction and a slightly null reduction in intermediate and high GEI. The variation in accuracy between years was more visible for the scheme based on with-cohort prediction.

### 3.3 Evolution of the genetic variance and the frequency of favorable alleles

The evolution in genetic variance showed an important depletion over the 30 years in all breeding schemes (**Figure 3**). The decline of the variance over the cycles was linked to the GEI levels, with a faster decline in the absence of GEI (**Supplementary Figure S4**; **Supplementary Table S2**). In addition, the initial genetic variance was higher when the GEI levels were intermediate and high. However, a big difference was observed between the genomic selection schemes and the baseline based on phenotypic selection. The variance decrease was much less severe in the baseline scenario, irrespective of the GEI level, with a decrease of 16%, 22%, and 35% relative to the base population genetic variance after 30 years of breeding, when GEI levels were 0, 4, and 8 respectively.

In contrast, the GS schemes showed a faster decline in genetic variance over the breeding cycles. The reduction of the genetic variance over the 30 breeding years was more important in the 2-Year-WP scheme in all GEI levels followed by the 3-Year scheme. The 2-Year-BP scheme showed a depletion pattern slightly similar to the 5-Year pattern, especially at intermediate and high levels of GEI. Additionally, the variance of the GS schemes was more severe during the first 15 years. The 2-Year-WP scheme gave a reduction of the genetic variance relative to the base population variance of 75%, 69%, and 71% during the first 15 years when the GEI levels were 0, 4, and 8, respectively. With regards to the 2-Year-BP, the depletion of genetic variance in the medium-term was less severe compared to the 2-Year-WP. For GEI levels of 0, 4, and 8, a relative decrease of the 2-Year-BP genetic variance compared to the base population variance was observed at 55%, 43%, and 45%, respectively.

**Figure 4:**
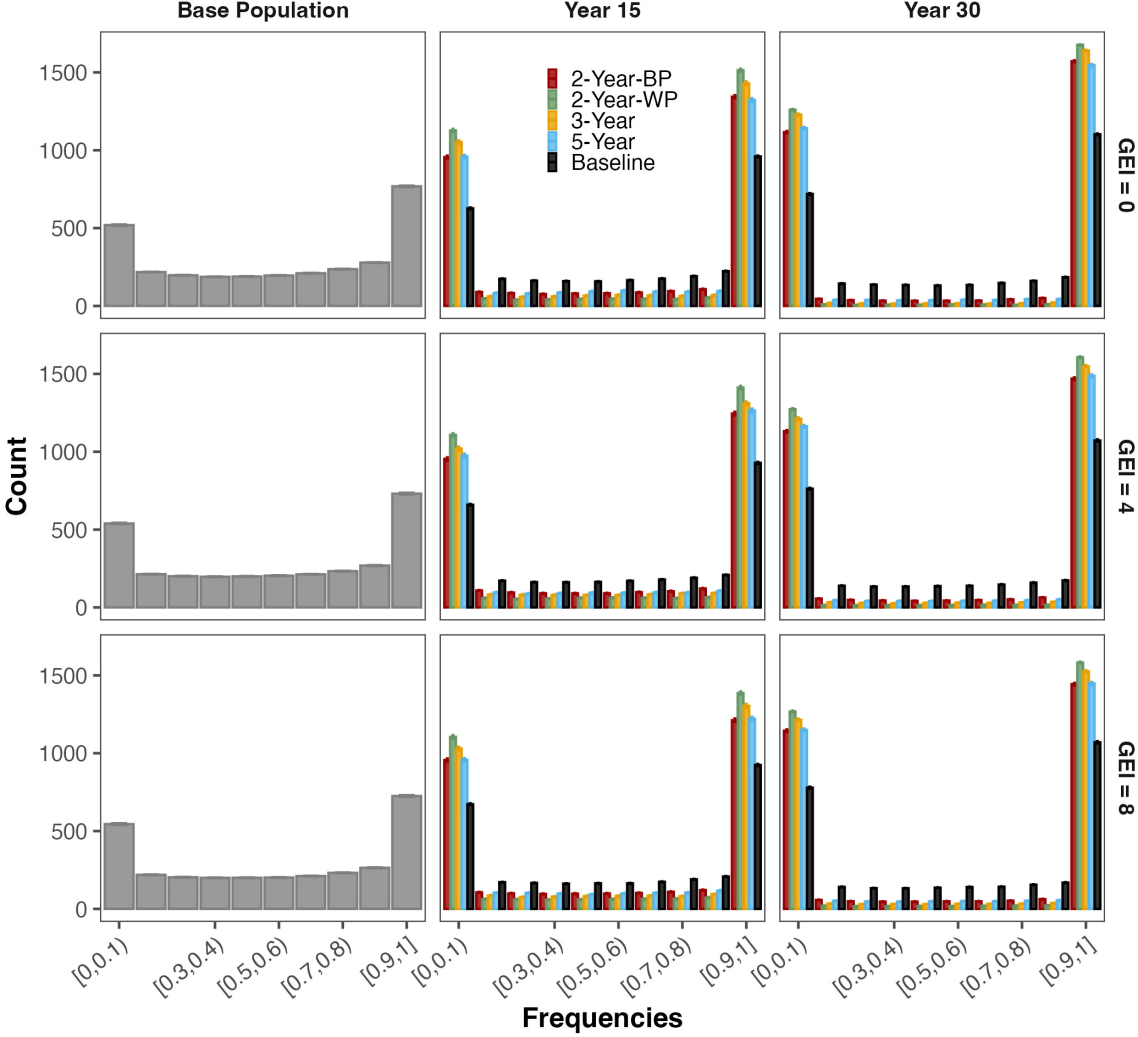
Evolution of favorable allele frequencies for each simulated QTL (3000) from the base population to the medium-term (Year 15) and long-term (Year 30). The four breeding schemes and the Baseline are presented, as well as the three levels of genotype-by-environment interaction (GEI) variances: 0 (top panel), 4 (middle panel), and 8 (bottom panel).

This decrease in the genetic variance was associated with the fixation or loss of favorable alleles for the 3000 simulated QTLs over the years (**Figure 4**; **Table 4**; **Supplementary Figure S5**) Initially, in the base population, 25% of the 3000 favorable alleles were nearly fixed (frequency greater than 0.90), whereas 18% were in very low frequency (lower than 0.10). For all schemes, the level of fixation or loss of favorable alleles was slightly higher in the absence of GEI (**Supplementary Figure S5**). The level of fixation was the lowest for the baseline, with a maximum of 29% of the favorable alleles fixed after 30 years. Among the GS schemes, the 2-Year-WP showed the highest degree of fixation and loss in both the medium- and long-term. In the medium term, from 43 to 47% of all the favorable alleles were fixed in the population depending on the level of GEI. These frequencies reached 51 to 55% in the long term. A similar pattern was observed for the other GS schemes but with a lower degree of fixation or loss of favorable alleles (**Table 4**). The 3-Year scheme presented an intermediate level of fixation compared to the 2-Year-WP and 2-Year-BP, with frequencies ranging from 39 to 44% and from 49 to 53% for the medium- and long-term, respectively. For the 2-Year-BP, the degree of fixation ranged from 35 to 39% in the medium-term and from 45 to 49% in the long term. Interestingly, for the GS schemes, the proportion of fixed favorable alleles in the long term was consistently 9 to 11% higher than those lost for intermediate and high GEI levels (**Figure 4**; **Table 4**). This is in contrast with the baseline, for which the proportion of favorable alleles fixed in the long term was higher by 7 to 8% compared to those lost at the same time.

**Table 4:** Percentage of Quantitative Trait Loci (QTLs) with the favorable allele fixed (frequency = 1) or lost (frequency = 0) at the two time horizons.

Scheme	GEI	Medium-Term (15 years)		Long-Term (30 years)	
		Lost (0)	Fixed (1)	Lost (0)	Fixed (1)
2-Year-BP	0	28	39	35	49
	4	28	36	35	46
	8	28	35	36	45
2-Year-WP	0	35	47	41	55
	4	34	43	41	52
	8	34	43	41	51
3-Year	0	32	44	40	53
	4	31	39	39	50
	8	31	39	39	49
5-Year	0	29	39	36	49
	4	28	36	37	47
	8	28	35	36	45
Baseline	0	16	23	20	29
	4	17	22	21	29
	8	17	22	22	29

The four breeding schemes, the Baseline, and the three levels of genotype-by-environment interaction (GEI) variance are presented. The total number of simulated QTLs was 3000.

## 4 Discussion

### 4.1 Reducing breeding cycle time

In recent years, one focus of the IRRI breeding strategy has been on shortening the breeding cycle time to enhance genetic gain for grain yield. Initially, efforts have been made to speed up the fixation of segregating material. The RGA methodology enabled the breeding program to shorten the line development time and breeding cycle by at least two years (Collard et al., 2019, 2017). More recently, the breeding cycle time was further reduced by the integration of genomic selection (Bartholomé et al., 2022). This reduction in cycle time has been recognized as beneficial for boosting the rate of genetic gain. Indeed, among the variables in the breeder’s equation, reducing the time required to complete the breeding cycle is the most straightforward approach to increasing rates of genetic gain in plant breeding programs (Atlin and Econopouly, 2022; Cobb et al., 2019; Sinha et al., 2021). Although reducing the generation interval through genomic selection has shown potential for higher genetic gain compared to phenotypic selection programs (Biswas et al., 2023; Gaynor et al., 2017; Heffner et al., 2010; Lubanga et al., 2023; Tessema et al., 2020; Werner et al., 2023), its effectiveness is amplified when combined with speed breeding techniques (Collard et al., 2017; Kabade et al., 2024; Li et al., 2015).

In the present study, we used simulation experiments for a data-driven optimization of the breeding program. We found that the relative increase in genetic gain was even more pronounced for shorter breeding cycles, especially when considering the medium-term. For example, reducing the breeding cycle from three years to two years resulted in a 27% increase in genetic gain, while shortening the cycle from five years to three years resulted in a 16% gain (**Table 3**). This difference suggests a non-linear relationship between genetic gain and breeding cycle time. According to the breeder’s equation and assuming that the selection accuracy, additive genetic variance, and selection intensity are unchanged and equal to 0.4, 1, and 1.75 respectively, reducing the breeding cycle from five to three years would increase the gain by 64.3% (**Supplementary Figure S6**). A breeding cycle reduction from three to two years would increase the gain by 52%. A reduction from a two- to a one-year cycle can increase the gain by 100% (**Supplementary Figure S6**). The impact of reducing the duration by one year on genetic gain is theoretically small for breeding programs with a long breeding cycle (8-10 years), but it becomes increasingly important when the breeding cycle is short (3 years or less). This pattern reflects a law of increasing returns, where the additional benefits of shorter breeding cycles become progressively larger. The continuous efforts at IRRI to reduce the length of the breeding cycle are supported by the results of the 2 years recycling schemes. However, achieving further reductions in future breeding strategies will require adopting a new and more efficient speed breeding protocol (Kabade et al., 2024).

### 4.2 Accuracy of predictions

The breeding strategies using the within-cohort prediction had the highest rates of genetic gain in the intermediate and high levels of GEI (**Figure 3**; **Supplementary Figure S3**). These higher rates of genetic gain are mainly related to the difference in the prediction accuracy between the within-cohort and the between-cohort predictions. The within-cohort prediction schemes showed the highest prediction accuracies in all breeding scenarios. Prediction accuracies are affected by several factors including the genetic architecture and heritability of the trait, the training population size, the genetic relationships between training and validation populations, and marker density (Ahmadi et al., 2020; Asoro et al., 2011; Heffner et al., 2011; Lorenz et al., 2012; Sallam et al., 2015; Spindel and Iwata, 2018; Zhong et al., 2009). According to the relationship mentioned above between cycle length and genetic gain, it is more effective to shorten the breeding cycle as much as possible. Shorter cycles can significantly increase the rate of genetic gain if prediction accuracies are maintained at a satisfactory level. The prediction accuracy decreases when relationships between the individuals in the training set and the selection candidates decrease (Lorenz, 2013; Lorenz and Smith, 2015). Also, higher broad-sense and narrow-sense heritabilities are associated with higher prediction accuracy (Kaler et al., 2022). In this study, only trait heritability and genetic relationships between training and validation sets are not fixed parameters between schemes. In the within-cohort prediction schemes, the training and the validation sets come from the same population, unlike the between-cohort prediction scheme, where genotypes from previous cohorts are combined to predict genotypes from the new cohort. The prediction accuracy of the 2-Year-BP initially increased, then leveled off, and finally declined continuously. Improving the genetic gain in the 2-Year-BP could be achieved by optimizing marker density and updating the training data using an optimization algorithm for selection, rather than selecting the most recent cohorts in the training population (Akdemir, 2014; Akdemir et al., 2021, 2015; Akdemir and Isidro-Sánchez, 2019; Kadam et al., 2021; Neyhart et al., 2017; Rincent et al., 2012; Sallam et al., 2015).

In another significant finding, the performance ranking of the 2-Year-BP scheme changed as the severity level of the GEI increased (**Figure 2**; **Supplementary Figures S1**, **S2**). The 2-Year-BP scheme showed a decrease in genetic gain of around 47% in the medium-term horizon from no to high GEI. The accuracy in intermediate and high GEI did not exceed 0.32 and 0.24, respectively. In contrast, accuracies of the within-cohort schemes reached a maximum of 0.43 and 0.41, respectively, for intermediate and high GEI. These results suggest that the between-cohort prediction is more sensitive to environmental variability. The relatively low prediction accuracy in moderate and high GEI in this study could be the result of a lack of connectivity between environments, mainly in the case of the between-cohort prediction scenario (2-Year-BP). Indeed, a high GEI leads to a loss of predictive ability of the genomic prediction model due to a lack of connectivity between environments (similarities among environments) and between genotypes (similarities among genotypes). As we know, a lack of correlation between environments is one of the main characteristics of GEI (Bustos-Korts et al., 2018; Malosetti et al., 2013). When the pairwise correlation between environments is negligible or negative, the observed performance of a set of genotypes in one environment may be unrelated to performance of the same genotypes or their relatives in another environment (Malosetti et al., 2013). In addition to the training set optimization described above, incorporating GEI and explicit environmental covariables in the prediction model could further improve the predictive ability in the 2-Year-BP scenario as demonstrated in many studies (Burgueño et al., 2012; Lopez-Cruz et al., 2015; Malosetti et al., 2016). However, crop scientists should take this result with caution and not jump to the conclusion that 2-Year-BP is highly sensitive to GEI. In this study, the modeling of the GEI was relatively simple due to the GEI models available in the tool, which generated a single phenotype for each genotype, encompassing a main effect, an interaction effect, and an error across environments. Thus, further research is needed to investigate the performance of 2-Year-BP with a more realistic GEI modeling. Using a compound symmetry GEI or, even better, a more realistic unstructured GEI could give us a better understanding of how GEI affects between-cohort prediction (Bančič et al., 2024; Werner et al., 2024).

### 4.3 Importance of GEI and its implication for testing strategy

The target population of environments (TPE) represents the set of farms and future seasons (soil quality, drainage, temperature, rainfall, daylight, diseases, etc.) in which the varieties produced by a breeding program will be grown. These environmental factors will vary considerably due to climate change, making predicting the future environment less accurate. Thus, one can expect that the GEI levels will increase for a targeted region. To account for this, we set the variance levels associated with GEI to be four and eight times higher than the genotype main effect variance. Currently, the observed proportion of GEI in the program ranges from 0.7 to around 0.9, depending on the region and the year. This relatively low level of GEI is advantageous for implementing methodologies such as sparse testing, allowing us to make the most of genotyping information in genomic prediction. An effective sparse-testing MET design might help to save considerable operational and financial resources while guaranteeing an optimal level of prediction accuracy, particularly in the case of the 2-Year-WP scenario where seed amplification may be compromised by lack of time. Indeed, the predictive ability of unobserved lines can be improved using genome-based prediction models encompassing the genotype x environment interaction. This approach results in unobserved genotype × environment combinations that can be better predicted, reducing the overall cost of MET trials (Atanda et al., 2022; Jarquin et al., 2020; Montesinos-López et al., 2023).

In the present study, the rate of genetic gain decreased significantly as the level of GEI increased in all the breeding schemes (**Figure 2**; **Table 2**). This effect of GEI on genetic gain partly results from the drop in prediction accuracy in intermediate and high GEI. However, it is interesting to note that, among the within-cohort prediction schemes, the shortest breeding cycle schemes showed a smaller decrease in genetic gain with increasing GEI. For example, from no GEI to high GEI levels, the 2-Year-WP, 3-Year, and 5-Year schemes displayed a reduction of genetic gains in the medium-term horizon of 19%, 22%, and 25%, respectively. This decrease in accuracy aligns with other genomic prediction studies involving GEI, using either simulation or empirical data (Bančič et al., 2024; Gaynor et al., 2017; Malosetti et al., 2016). This decrease in accuracy could be related to the intensity of environmental variability over the years resulting in genotype-by-year interaction. Thus, fast recycling would help to capture smaller environmental variations and develop genotypes that can adapt to short-term changes in the TPE, another advantage of going for shorter recycling schemes. However, further research is needed to investigate the impact of early recycling on GEI mitigation.

### 4.4 Practical implication for breeding

One component of breeding optimization is reducing the breeding cycle length to ultimately increase genetic gains. However, shorter breeding cycles must be achieved while minimizing costs. The 2-Year-WP offers a significant advantage for use in breeding programs as it is the most efficient in terms of realized gain and in cost-effectiveness. By reducing the RGA exit to the F3 generation, the 2-Year-WP decreases the RGA cost compared to the 3-Year scheme. Our results show that the 2-Year-WP costs around 8% less than the 3-Year scheme (**Supplementary Table S3**). On the other hand, the 2-Year-BP scheme shifts to predicting untested lines based on previous data, resulting in a cost comparable to the 3-Year scheme. While the 2-Year-WP scheme is the most effective and cost-efficient, based on the simulations, its practical implementation is quite challenging. The activities planned for the second year — RGA, seed amplification, genotyping, and Stage 1 evaluation — along with the associated time constraints might cause a delay in the establishment and evaluation of MET in the regions. The Stage 1 yield trial alone takes roughly 6 months to complete accurately at IRRI headquarters. The seed amplification step and post-harvest operations take more time. The fixation stage will need modifications to overcome constraints and take advantage of this strategy. Therefore, the crosses and the first generation of the RGA will have to be completed in Year 1, using speed breeding protocols. Given the constraints associated with seed shipment, more time will be needed for the Stage 1 evaluation in regions to ensure data availability.

## 5 Conclusion

The most effective way to significantly increase genetic improvement is by shortening the time it takes to complete a breeding cycle. In this study, we found that shortening the cycle from three years to two years using a within-cohort prediction led to a genetic gain increase up to 27% in the medium term. However, using a between-cohort prediction scheme to reduce the breeding cycle could reduce breeding efficiency, especially in the presence of high genotype-by-environment interactions. Therefore, for aggressive breeding programs that already have a short cycle length, further reducing the cycle through between-cohort genomic selection could decrease the rate of genetic gain due to lower prediction accuracy. When a breeding program adopts a 2-Year within-cohort scheme, there is very little time for crossing, evaluation, and selection processes. Therefore, to implement this scheme efficiently, the breeding operations process should include quick seed shipment and clearance, as well as a swift turnaround time for trial data from partners. Meanwhile, the 2-Year between-cohort prediction scheme with an optimized training set could be a good alternative to achieve higher genetic gain when the GEI in a breeding pipeline is low.

## Data Availability

All the breeding simulation source codes used in this study are available from the GitHub repository: https://github.com/fallseck/IRRI-OneRice-Simulation.

## In memoriam

This work is dedicated to Professor Tala Gueye, one of the co−authors, who passed away before the publication of this work. It is heart−wrenching to know that he will not be around to witness the fruits of his hard work and collaboration. Professor Gueye was a wonderful person, so much valued by everyone who knew him, and a great colleague. His availability and helpful nature were just a few of his qualities that we all appreciated. We are grateful for all the moments shared, the work done, and the shared memories. His presence will be missed dearly. May his soul rest in peace.
