# Supplementary material for: Stochastic simulation to optimize rice breeding at IRRI
Source: Front Plant Sci. 2024 Nov 1;15:1488814. doi: 10.3389/fpls.2024.1488814 (PMC11563958; doi:10.3389/fpls.2024.1488814)
Supplement: Supplementary file 1 [file DataSheet1.pdf]

Supplementary material

Stochastic simulation to optimize rice breeding at IRRI.

Fallou Seck[1,2], Parthiban Thathapalli Prakash[1], Giovanny Covarrubias-Pazaran[1], Tala Gueye[2], Ibrahima Diédhiou[2], Suresh Kadaru[1], Jérôme Bartholomé[3,4,5]

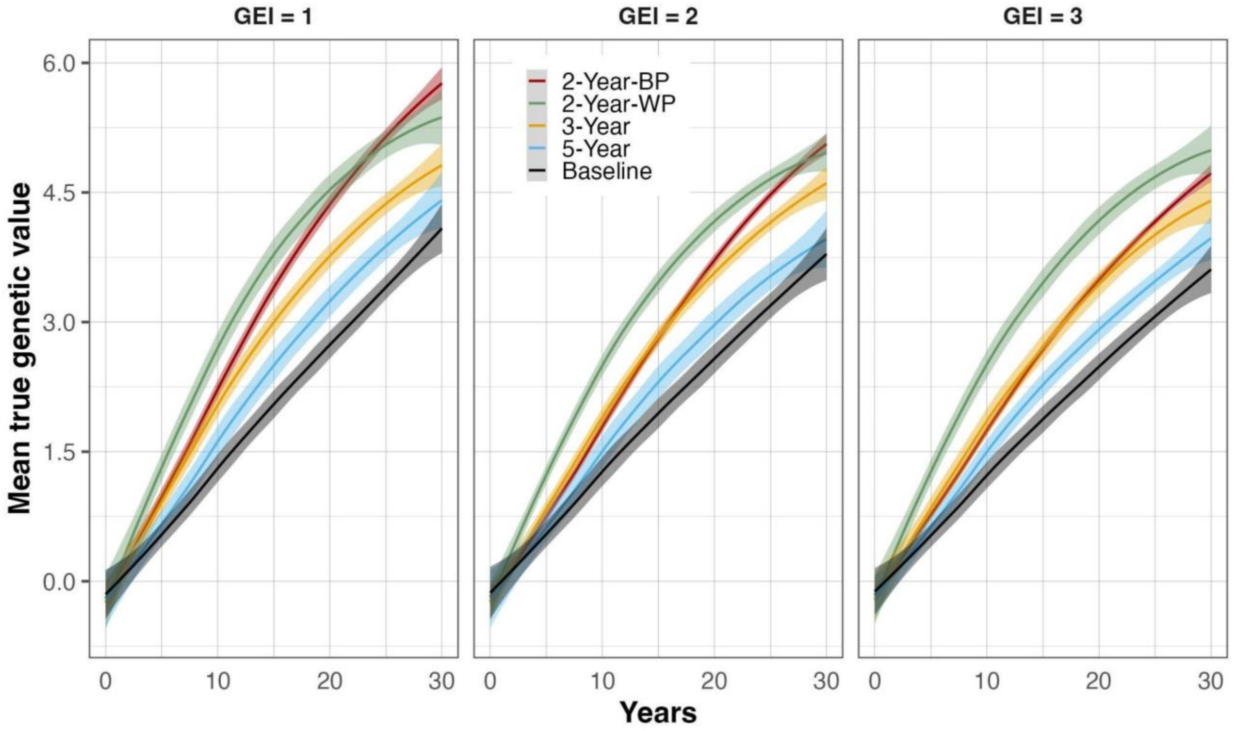

Figure S1: True genetic value trends at the parent-recycling stage over 30 breeding years of all the breeding schemes when genotype-by-environment (GEI) variances were 1 (left panel), 2 (middle panel), and 3 (right panel) times greater than the main genetic variance. The breeding schemes are represented in colored lines: the 5-Year parent recycling scheme (5-Year), the 3-Year parent recycling scheme (3-Year), the 2-Year between-cohort prediction recycling scheme (2-Year-BP), and the 2-Year within-cohort prediction recycling scheme (2-Year-WP). The solid lines represent the average value, and the shaded areas represent the associated standard error based on 100 replicates for each scenario.

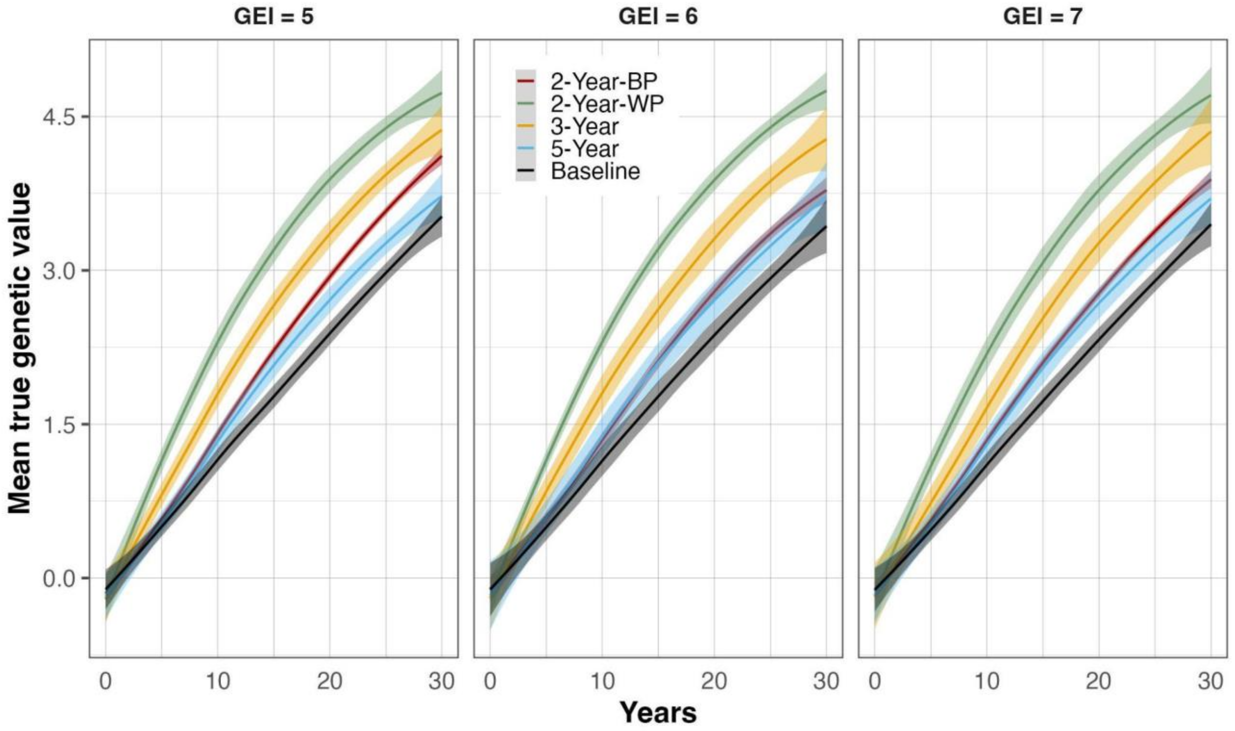

Figure S2: True genetic value trends at the parent-recycling stage over 30 breeding years of all the breeding schemes when genotype-by-environment (GEI) variances were 5 (left panel), 6 (middle panel), and 7 (right panel) times greater than the main genetic variance. The breeding schemes are represented in colored lines: the 5-Year parent recycling scheme (5-Year), the 3-Year parent recycling scheme (3-Year), the 2-Year between-cohort prediction recycling scheme (2-Year-BP), and the 2-Year within-cohort prediction recycling scheme (2-Year-WP). The solid lines represent the average value, and the shaded areas represent the associated standard error based on 100 replicates for each scenario.

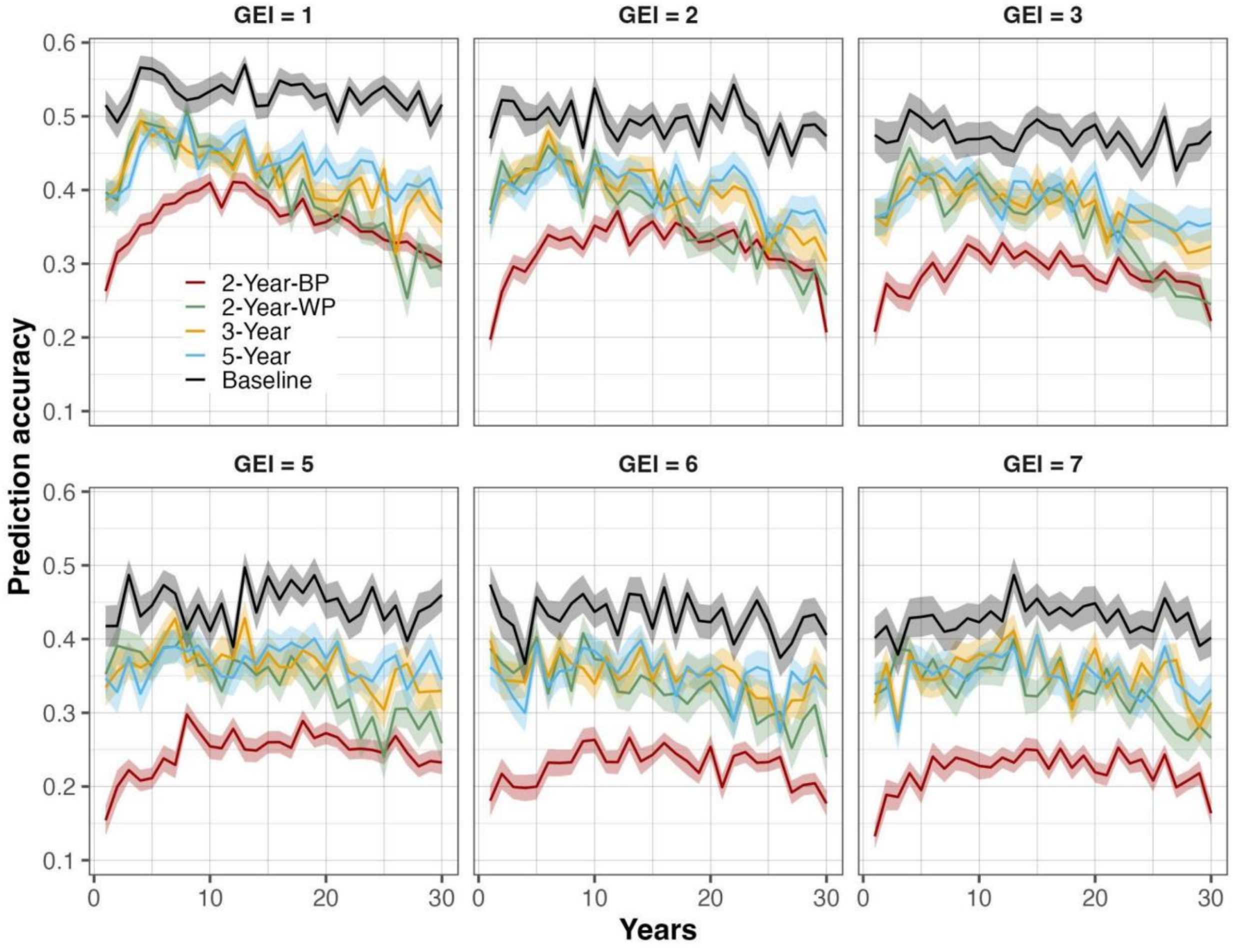

Figure S3: Genomic prediction accuracy at the parent-recycling stage over 30 breeding years for all breeding schemes when genotype-by-environment (GEI) variances were: 1, 2, 3, 5, 6, and 7 times greater than the main genetic variance. The colored lines represent the different breeding schemes: the 5-Year parent recycling scheme (5-Year), 3-Year parent recycling scheme (3-Year), the 2-Year between-cohort prediction recycling scheme (2-Year-BP), and the 2-Year within-cohort prediction recycling scheme (2-Year-WP). The solid lines represent the average value, and the shaded areas represent the associated standard error based on 100 replicates for each scenario.

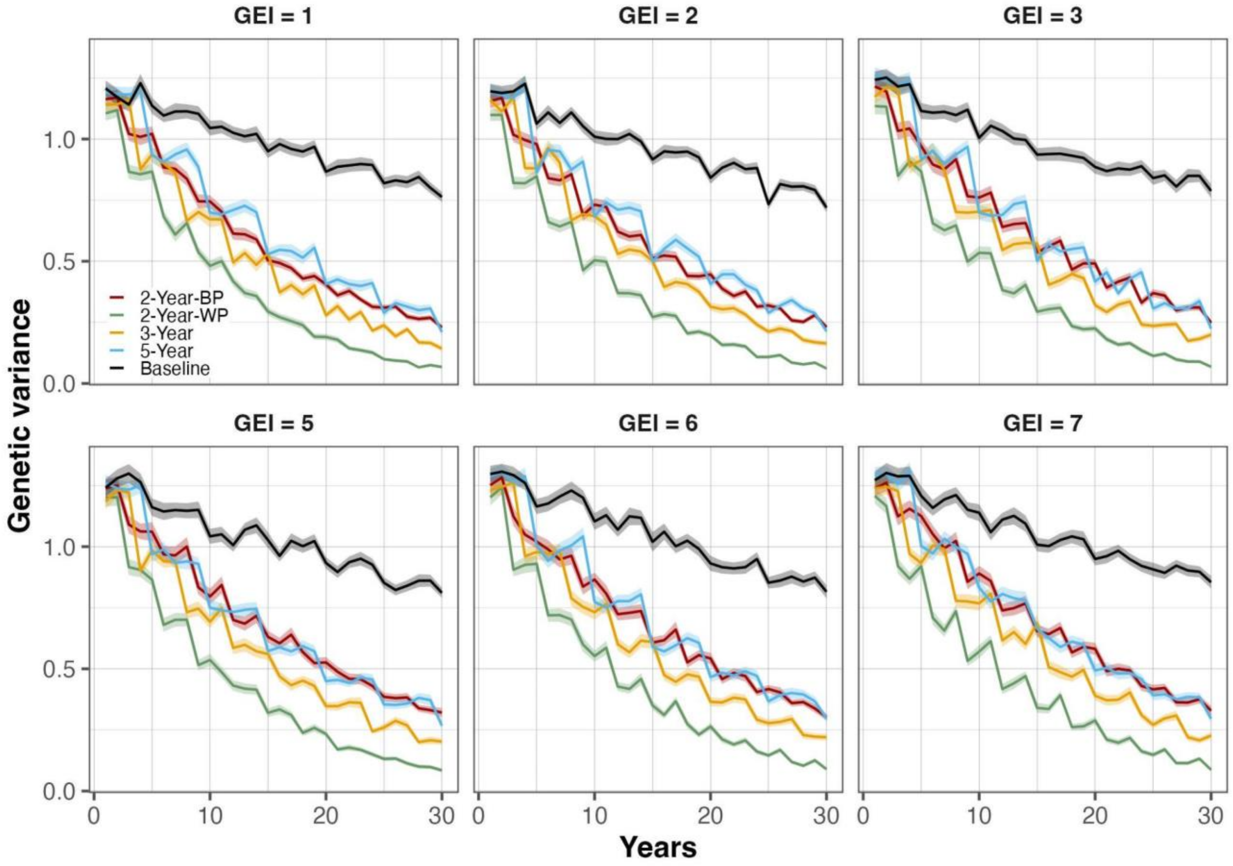

Figure S4: Genetic variance at the parent-recycling stage over 30 breeding years for all breeding schemes when genotype-by-environment (GEI) variances were: 1, 2, 3, 5, 6, and 7 times greater than the main genetic variance. The colored lines represent the different breeding schemes: the 5-Year parent recycling scheme (5-Year), the 3-Year parent recycling scheme (3-Year), the 2-Year between-cohort prediction recycling scheme (2-Year-BP), and the 2-Year within-cohort prediction recycling scheme (2-Year-WP). The solid lines represent the average value, and the shaded areas represent the associated standard error based on 100 replicates for each scenario.

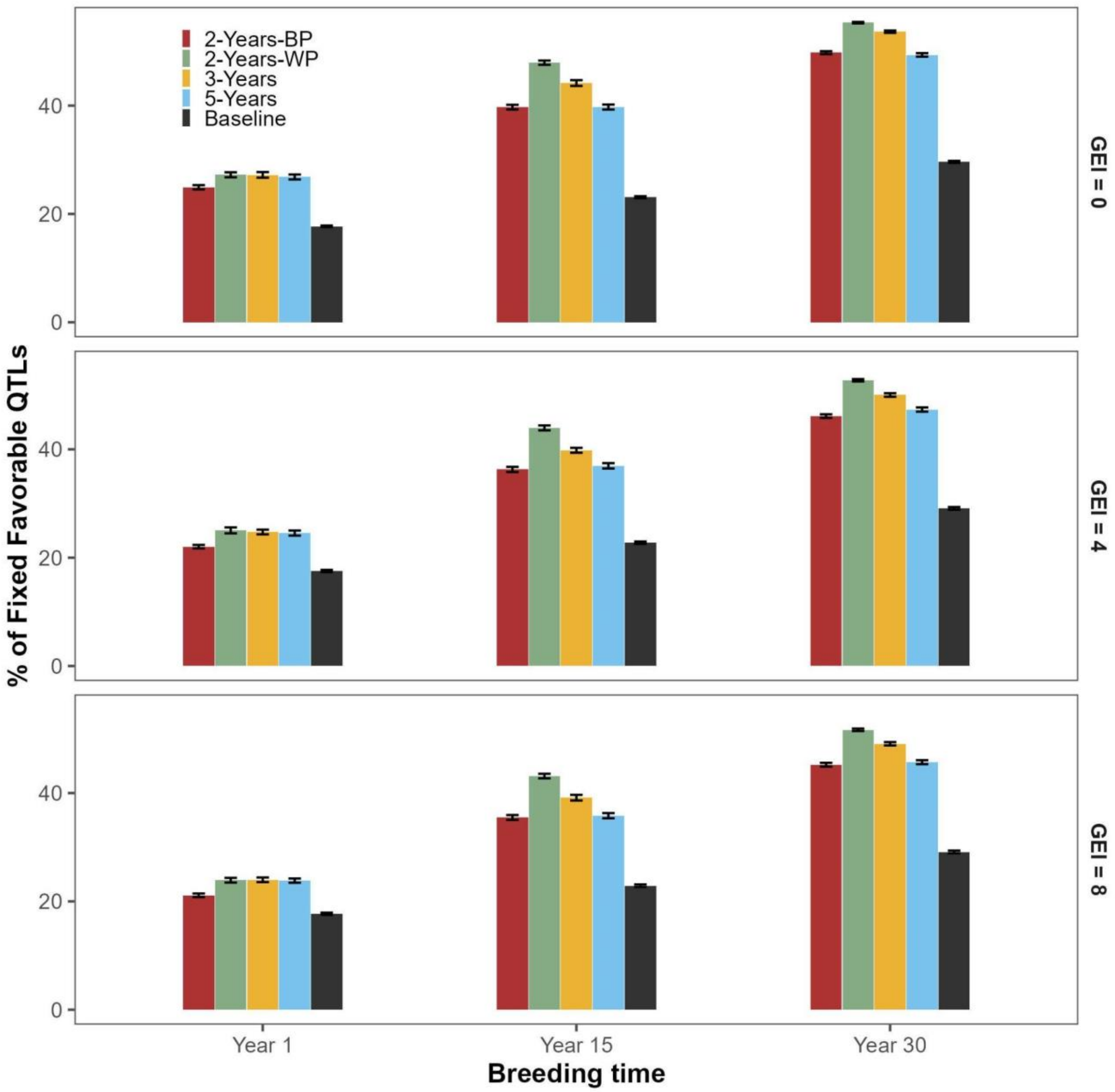

Figure S5: Percentage of favorable quantitative trait loci (QTLs) that are fixed in the entire 3000 simulated QTLs for the four evaluated breeding schemes in the first breeding cycle (Year 1), medium-term breeding (Year 15), and the long-term breeding (Year 30) when genotype-by-environment (GEI) variances were 0 (top panel), 4 (middle panel), and 8 (bottom panel)

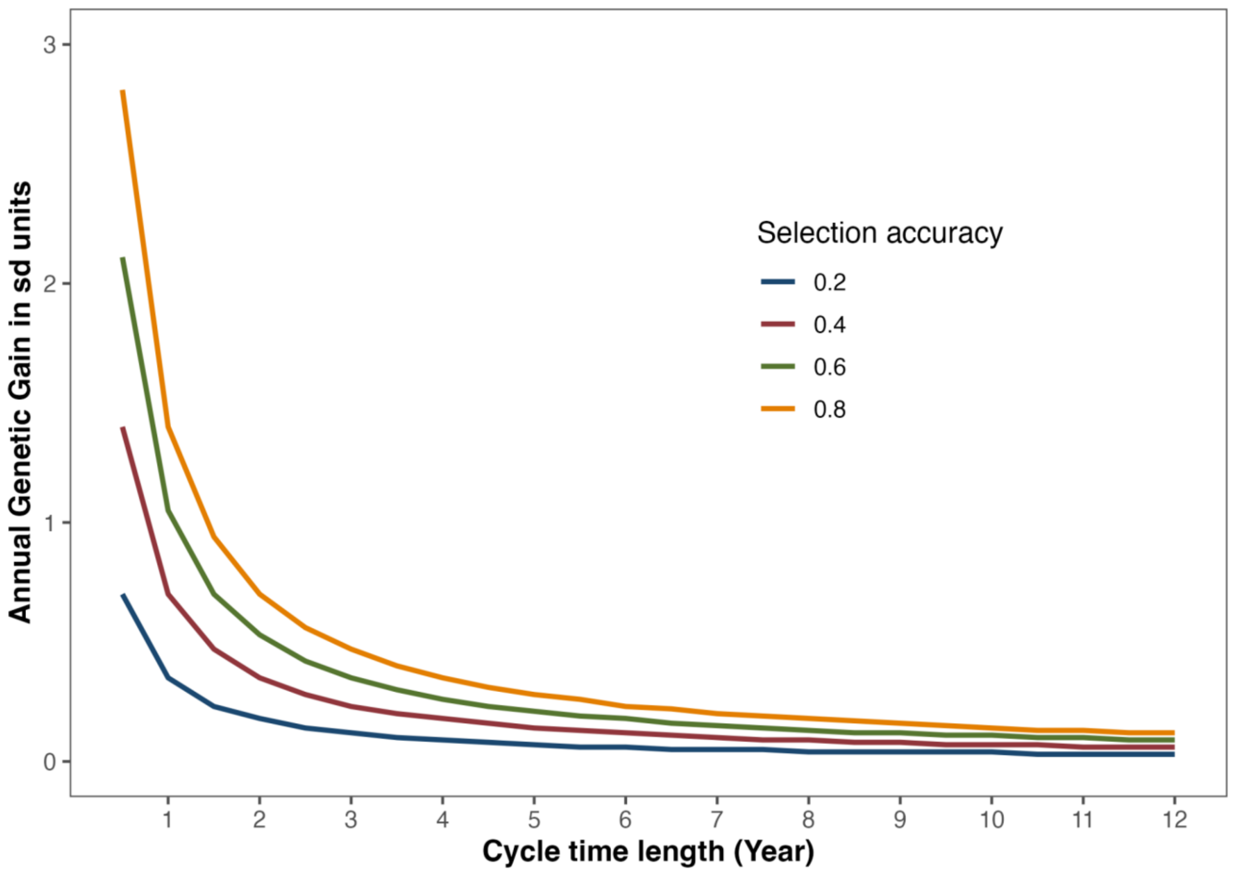

Figure S6: The expected rate of genetic gain per year with increasing levels of selection accuracy and breeding cycle length. Data are simulated from the breeder's equation with a fixed genetic variance of 1 and a selection intensity equal to 1.75. The relationship between the rate of genetic gain and the cycle length suggests a mathematical relationship of 1/x.

Table S1: Relative additive variance of the main effect in the total genetic variance according to genotype-environment variance (GEI).

Main Genetic variance	GEI variance	Relative Additive main effect
1	0	1.00
	1	0.50
	2	0.33
	3	0.25
	4	0.20
	5	0.17
	6	0.14
	7	0.13
	8	0.11

Table S2: Minimum (min), maximum (max), and mean of genetic variance and prediction accuracy for all four evaluated breeding schemes over the 30 breeding years. SE (Standard error)

GEI	Scenario	Metric	Min	Max	Mean	SE
0	2-Year-BP	Genetic variance	0.15	1.07	0.51	0.049
0	2-Year-WP	Genetic variance	0.04	1.04	0.35	0.055
0	3-Year	Genetic variance	0.11	1.08	0.47	0.055
0	5-Year	Genetic variance	0.15	1.11	0.58	0.053
0	Baseline	Genetic variance	0.71	1.12	0.90	0.021
4	2-Year-BP	Genetic variance	0.28	1.19	0.68	0.050
4	2-Year-WP	Genetic variance	0.09	1.17	0.46	0.061
4	3-Year	Genetic variance	0.18	1.22	0.60	0.058
4	5-Year	Genetic variance	0.29	1.24	0.71	0.055
4	Baseline	Genetic variance	0.82	1.25	1.02	0.023
8	2-Year-BP	Genetic variance	0.35	1.37	0.76	0.055
8	2-Year-WP	Genetic variance	0.10	1.30	0.51	0.066
8	3-Year	Genetic variance	0.23	1.35	0.67	0.064
8	5-Year	Genetic variance	0.29	1.38	0.78	0.060
8	Baseline	Genetic variance	0.82	1.42	1.11	0.030
0	2-Year-BP	Accuracy	0.32	0.49	0.43	0.010
0	2-Year-WP	Accuracy	0.27	0.57	0.43	0.017
0	3-Year	Accuracy	0.32	0.58	0.46	0.013
0	5-Year	Accuracy	0.34	0.55	0.49	0.008
0	Baseline	Accuracy	0.34	0.64	0.60	0.009
4	2-Year-BP	Accuracy	0.14	0.32	0.26	0.007
4	2-Year-WP	Accuracy	0.16	0.41	0.34	0.010
4	3-Year	Accuracy	0.16	0.42	0.36	0.009
4	5-Year	Accuracy	0.16	0.43	0.37	0.008

GEI	Scenario	Metric	Min	Max	Mean	SE
4	Baseline	Accuracy	0.16	0.52	0.44	0.010
8	2-Year-BP	Accuracy	0.12	0.24	0.21	0.005
8	2-Year-WP	Accuracy	0.12	0.40	0.32	0.011
8	3-Year	Accuracy	0.12	0.39	0.34	0.009
8	5-Year	Accuracy	0.12	0.41	0.35	0.009
8	Baseline	Accuracy	0.12	0.50	0.42	0.011

Table S3: Estimated budgets in USD of the 3-Year, 2-Year-BP, and 2-Year-WP schemes.

Years	Seasons	Activities	Size	Unit price (USD)	Generations	3-Year and 2-Year-BP	Generations	2-Year-WP
1	S1	Crosses	30	27.54	Parents	826.2	Parents	826.2
1	S1	Genotyping	450	4.12	Parents	1854	Parents	1854
1	S2	F1	30	29.32	F1	879.6	F1	879.6
1	S2	Genotyping	150	4.12	F1	618	F1	618
2	S1	RGA	7200	1.54	F2 - F4	11088	F2 - F3	5544
2	S1	Genotyping	7200	4.12	F2	29664	F2	29664
2	S2	SA	1200	1.7	F4 - F5	2040	F3 - F4	2040
2	S2	Genotyping	1200	15.58		18696		18696
3	S1	STAGE1	300	11.1	F5 - F6	3330	F4 - F5	3330
3	S2							
4	S1	STAGE2	40	26.08	F6 - F7	1043.2	F5 - F6	1043.2
4	S2							
**Total (USD)**						**70039**		**64495**
**%Change**						**7.92%**		
